# A comprehensive review on self-cleaning glass surfaces: durability, mechanisms, and functional applications

**DOI:** 10.1039/d4ra06680d

**Published:** 2024-10-28

**Authors:** Suqi Xue, Shanglei Yang, Xiner Li, Qiubo Li, Bangguo Hu

**Affiliations:** a School of Materials Science and Engineering, Shanghai University of Engineering Science Shanghai 201620 China yslei@sues.edu.cn xxueqii@163.com Lxe200408@163.com liqiubo2023@163.com hubg2023@163.com; b Shanghai Laser Intelligent Manufacturing and Quality Inspection Professional Technical Service Platform Shanghai 201620 China

## Abstract

Self-cleaning glass surfaces have emerged as a focal point in the field of materials science due to their potential to reduce the accumulation of pollutants, enhance transparency, and improve durability. In recent years, significant advancements have been made in self-cleaning technologies based on photocatalysis and wettability regulation, particularly in the development of superhydrophobic and superhydrophilic surfaces. This article provides a systematic review of the research progress in self-cleaning technologies for glass surfaces. It analyzes and summarizes the applicability of self-cleaning effects induced by special properties such as photocatalysis, superhydrophobicity, superhydrophilicity, and omniphobicity on glass surfaces. Subsequently, the article delves into a discussion of the durability of these surface treatment technologies in practical applications, especially their stability and long-term performance under harsh environmental conditions. Furthermore, the paper explores the current status of applications for self-cleaning glass surfaces across various fields and proposes potential solutions and future research directions to address the challenges encountered in the practical application of self-cleaning glass surfaces.

## Abstract 2

With the advancement of materials engineering and the coatings industry, glass with excellent self-cleaning properties has emerged as a prominent research area.

## Graphical abstract

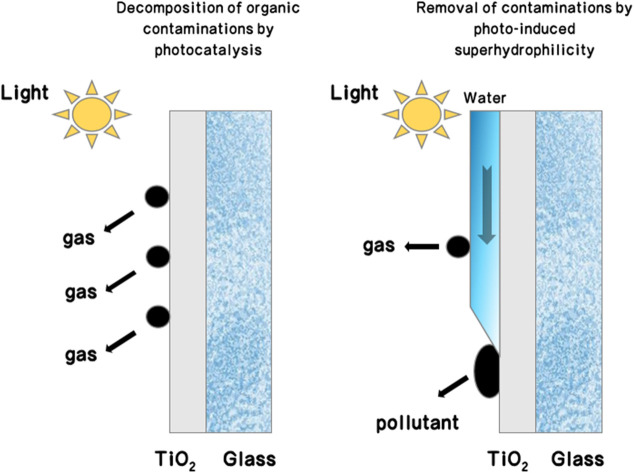

## 1 Introduction

As an important industrial material, glass is widely used in various fields such as construction, automobiles, photovoltaics, electronics and daily utensils. However, glass surfaces are susceptible to contamination, scratching, and corrosion, which can diminish their optical transparency, service life, and aesthetics. With technological advancements, scientists and engineers have been endowing glass surfaces with special properties like self-cleaning and durability through functional surface treatments, thereby expanding the application fields of glass and enhancing its value.^1,2^ Among these surface treatment technologies, self-cleaning glass has garnered widespread attention and research due to its effectiveness in resisting the accumulation of pollutants and maintaining long-term cleanliness.

Research on self-cleaning surfaces dates back to the natural phenomena known as the “Lotus effect” and the “Photocatalytic effect”. The Lotus effect is characterized by superhydrophobicity, enabling water droplets to form a high contact angle on the surface, and roll off while carrying away dust and other contaminants. In contrast, the Photocatalytic effect facilitates the degradation of pollutants on the surface through the catalytic action of ultraviolet radiation, leading to their automatic decomposition.^3,4^ Inspired by these phenomena, researchers have developed various self-cleaning glass surface treatment technologies with biomimetic design and the application of nanomaterials, such as superhydrophobic coatings and photocatalytic films. These technologies enable glass to maintain cleanliness automatically under the action of rainwater or sunlight, reducing reliance on traditional cleaning methods. Currently, research on self-cleaning glass surfaces primarily focuses on two aspects: one is the self-cleaning surfaces based on the photocatalytic principle, and the other is the self-cleaning surfaces based on the regulation of surface wettability.^4,5^ Photocatalytic self-cleaning surfaces utilize photocatalysts to produce strong oxidizing substances under light exposure, breaking down surface contaminants into carbon dioxide and water, thus achieving self-cleaning. Wettability-based self-cleaning surfaces, on the other hand, regulate the surface's wettability to render it superhydrophilic or superhydrophobic, using the spread or roll of water droplets to remove surface contaminants.

However, merely possessing self-cleaning properties is insufficient to meet the diverse demands of practical applications. The durability of glass surfaces is also a critical issue, especially under harsh conditions such as outdoor exposure, acid rain erosion, high temperatures, and mechanical wear, where the functional surface of the glass may be significantly compromised.^6,7^ Therefore, enhancing the long-term stability and durability of self-cleaning glass has become a focal point of research. Moreover, different application scenarios impose varying functional requirements on glass; for instance, photovoltaic glass demands high light transmittance and self-cleaning surfaces to improve the efficiency of solar energy conversion, while automotive glass focuses more on multiple properties such as water resistance, anti-fog, and scratch resistance.^8,9^ Consequently, the development of self-cleaning surface materials and treatment processes tailored to specific application needs has also become a hot topic of research.

This review aims to systematically recount the advancements in the research of self-cleaning glass surfaces, with a particular focus on the principles of self-cleaning technology, surface modification methods, durability issues, and their functional performance in practical applications. Through the analysis and summarization of existing literature, this article will outline the current status and challenges of self-cleaning glass surface applications across various fields and look forward to future development directions. Self-cleaning glass surface technology has not only brought convenience to modern industry and daily life but also provided significant technological support for environmental protection and sustainable development. Thus, in-depth research and optimization of this technology hold broad application prospects and economic benefits.

## 2 Various self-cleaning surfaces of glass

The self-cleaning effect can be induced through photocatalysis and special wettability, which can spontaneously clean contaminants adhering to its surface in various ways. By coating a nanocrystalline TiO_2_ film on the glass surface and activating the photocatalytic process with solar radiation, organic pollutants adhering to the surface can be effectively purified, thus achieving self-cleaning. For instance, Activ™ developed by Pilkington glass represents the first example of self-cleaning glass, composed of a nanocrystalline TiO_2_ film approximately 15 nm thick deposited on the glass surface.^5^ This product possesses high photocatalytic activity, strong durability, and features low visible light reflectance and favorable absorption of solar radiation, making it suitable for a variety of self-cleaning applications. Concurrently, research on biomimetic self-cleaning surfaces with special wettability, inspired by various natural phenomena such as the lotus leaf,^7^ rose petals,^8^ water strider legs,^9^ butterfly wings,^10^ shark skin,^11^ and the surface of the pitcher plant,^12^ is also steadily advancing. The synergistic effect of combining photocatalysis and special wettability induces a self-cleaning effect that can more effectively remove various surface contaminants.

### 2.1. Self-cleaning surfaces induced by photocatalysis

**Fig. 1:**
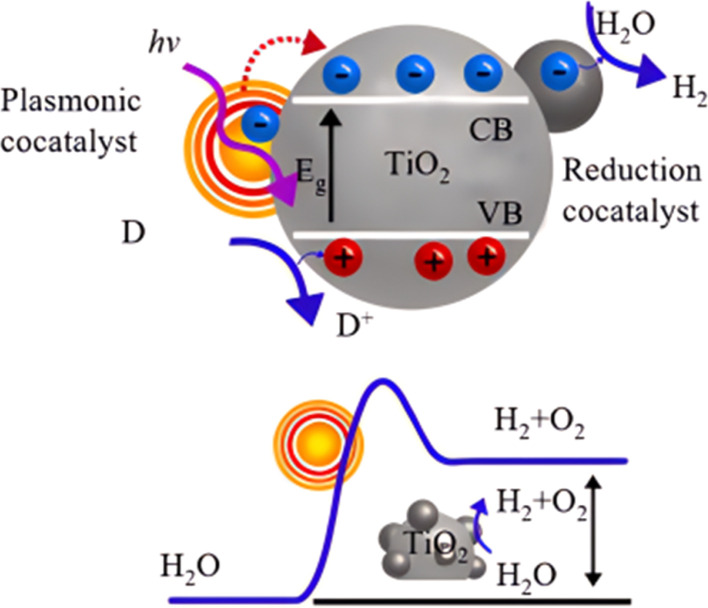
Principle of photocatalytic oxidation of TiO_2_ nanoparticles.^14^

**Fig. 2:**
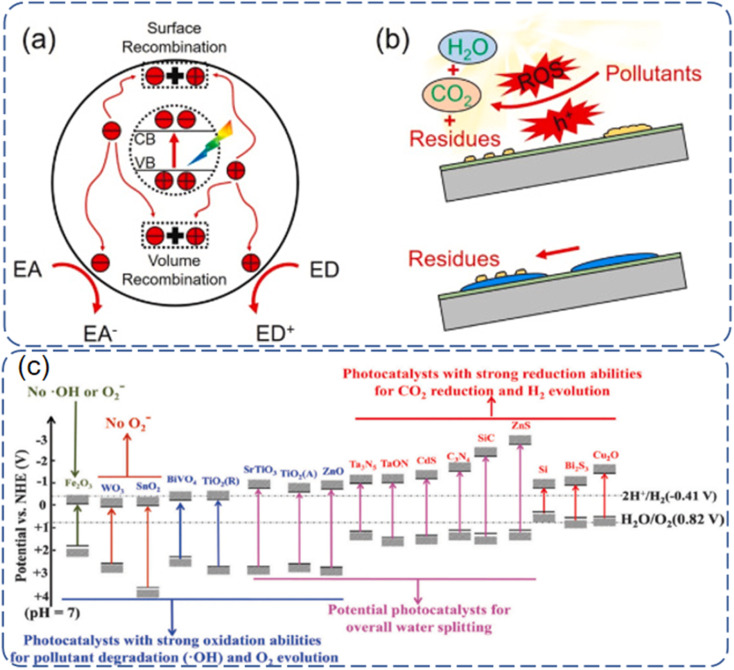
Schematic illustration of (a) photoexcited charges in with possible action pathways and (b) photocatalytic self-cleaning principle.^15^ (c) Conduction band and valence band values of some typical photocatalysts.^16^

Photocatalytic induced self-cleaning surfaces refer to the process of utilizing photocatalytically active materials, such as TiO_2_, to generate charge carriers under the influence of light, thereby achieving the degradation and cleaning of surface contaminants.^13^ A schematic illustration of TiO_2_ photocatalysis is depicted in Fig. 1.^14^ Under ultraviolet (UV) irradiation, electrons in the valence band of TiO_2_ are excited, creating photogenerated electrons and holes. Although these photogenerated electrons and holes are highly reactive, their short lifetimes limit their practical applications. These charge carriers can migrate to the surface of the catalyst, where they may be captured by lattice defects, react with other substances on the catalyst surface, or decompose at high temperatures, leading to a loss of activity. The photogenerated holes possess a high redox potential, making them excellent oxidizing agents capable of reacting with organic compounds to produce more electrons, thus accelerating the degradation of pollutants. It should be noted that photocatalysts are chemically active only if the photo-induced electron–hole pairs are consumed within nanoseconds to prevent their recombination.^15^ The excitation and formation of electron–hole pairs in photocatalysts are also very rapid, and they undergo various processes such as separation, migration, and recombination under light illumination (Fig. 2a). The principle of photocatalytic self-cleaning typically involves the combination of two processes: (i) the photocatalytic degradation of adsorbed contaminants by ROS and H^+^ produced by light, and (ii) the washing away of degraded residues by water through (super)hydrophilicity induced by light (Fig. 2b). Furthermore, the thermodynamic driving force of photocatalytic reactions largely depends on the potentials of the conduction band (CB) and valence band (VB) of the semiconductor catalyst, as well as the redox potential.^16^ The conduction and valence band values of typical photocatalysts are shown in Fig. 2c. TiO_2_ has unique positions of its conduction and valence bands, endowing it with excellent photocatalytic oxidation and reduction capabilities. Once excited, it readily forms strong oxidizing agents, such as electron–hole pairs, ˙OH, ˙O_2_− radicals, and H_2_O_2_.

There are generally two common methods for preparing photocatalytic self-cleaning surfaces on glass. One effective method is to coat the glass surface with a nanocrystalline TiO_2_ film, using solar radiation to activate the photocatalytic process and achieve self-cleaning ability. Another method involves forming heterostructures of TiO_2_ with other materials to enhance charge separation and improve photocatalytic activity and self-cleaning efficiency.^17–19^ In addition, doping TiO_2_ with non-metal elements, especially nitrogen, can increase hydrophilicity and visible light-induced photocatalytic activity. The combination of surface roughness and heterostructures has also been found to be more effective in preparing photocatalytic self-cleaning materials.^5^

### 2.2. Self-cleaning surface caused by wettability

**Fig. 3:**
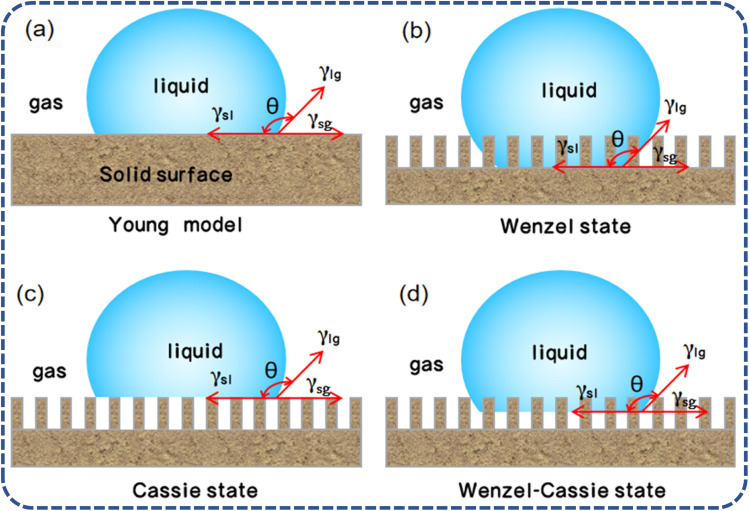
Contact models of water droplets on solid surfaces: (a) Young model; (b) Wenzel state; (c) Cassie state; (d) Wenzel–Cassie state.^21–26^

Inspired by natural phenomena such as the “lotus effect”, there has been rapid development in the field of wettability surface preparation in recent years. The wettability of glass surfaces is closely related to the chemical properties and microstructure of the materials, as well as the contact angle with various liquids.^20–23^ The so-called contact angle is the angle *θ* formed at the intersection of the solid–liquid and liquid–gas interfaces when a liquid drop comes into contact with a solid surface, establishing a three-phase contact line. When the solid, liquid, and gas phases reach a state of thermodynamic equilibrium, the angle formed at the boundary between the solid–liquid interface is known as the contact angle (CA). The wettability of a surface is classified based on the magnitude of the CA: if the CA is less than 90°, the surface of the solid material is designated as hydrophilic, with lower angles signifying a higher degree of hydrophilicity. A surface exhibits superhydrophilic properties when the CA is less than 10°. On the flip side, when the CA exceeds 90°, the surface is characterized as hydrophobic. Furthermore, the surface is said to enter a state of superhydrophobicity when the CA surpasses 150°, coupled with a contact angle hysteresis (CAH) of less than 5°. The wetting models originate from Young's equation (Fig. 3a), which was derived for sessile drops on an ideal rigid, uniform, flat, and inert surface. Young's equation^24^ posits that the contact angle on a sample surface is determined by the solid–liquid interfacial free energy (*γ*_SL_), the solid-vapor interfacial free energy (*γ*_SV_), and the liquid–vapor interfacial free energy (*γ*_LV_), with the corresponding equation given by:
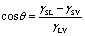


As the Young's equation is only applicable to ideal smooth surfaces and not practical in real life, Wenzel and Cassie built upon it to study the wettability of rough surface structures. The Wenzel model introduces the roughness factor (*r*), which is the ratio of the actual surface area to the geometric surface area. This factor is used to quantify the effect of surface roughness on wettability. The wettability equation for this rough solid surface is given by:
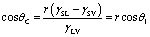
where *θ*_1_ and *θ*_C_ are the contact angles of smooth solid surface and actual solid surface, respectively. The Wenzel model^25^ posits that a rough surface increases the contact area between the liquid and the solid surface, thereby enhancing surface wettability. When the surface roughness comes into contact with a liquid, the droplet penetrates into the recesses of the rough structure. For hydrophilic surfaces, surface roughness can improve wettability; whereas for hydrophobic surfaces, surface roughness may reduce wettability and increase hydrophobicity.

However, the Wenzel model (Fig. 3b) assumes that the droplet completely wets the rough surface, without considering the role of air within the rough structure, and thus may not be entirely applicable in certain situations, such as on superhydrophobic surfaces with micro–nano structures. The Cassie model (Fig. 3c) offers a theoretical perspective that complements the Wenzel model, describing the wetting phenomenon when a droplet forms a heterogeneous wetting state on a solid with a rough surface. The Cassie model^26,27^ suggests that on a rough surface, the droplet does not completely wet the surface but rather makes contact on the peaks of the surface roughness, leaving air in the recesses, a state referred to as a heterogeneous wetting state or the Cassie state. The corresponding equation is:cos *θ*_C_ = *f*_1_ cos *θ*_1_ − *f*_2_In this equation, *f*_1_ and *f*_2_ represent the ratios of the solid area beneath the droplet to its projected area, and the ratio of the air area beneath the droplet to its projected area, respectively. The Cassie model elucidates that by introducing an appropriate level of surface roughness coupled with a reduction in surface energy, superhydrophobicity can be realized. This implies that the contact between the droplet and the surface is confined to the peaks of the rough structures, with air being trapped between the droplet and the remainder of the surface. Consequently, this arrangement diminishes the adhesive forces between the droplet and the surface, thereby enhancing its stability.

In practical applications, the transition between the Wenzel and Cassie models is a crucial concept.^21–23^ When the contact angle of a droplet is less than the critical contact angle (*θ*_C_), the surface tends to exhibit a Wenzel state, where the droplet fills all the surface recesses. Conversely, when the contact angle is greater than *θ*_C_, the surface tends to exhibit a Cassie state, where the droplet only contacts the top of the surface asperities. The Wenzel–Cassie model (Fig. 3d) represents a transitional state between these two extreme wetting states, thus by adjusting the surface's chemical composition and microstructure, one can control the wetting behavior of droplets on the surface, achieving various application goals.

Traditional glass possesses inherent polarity, which grants it a degree of hydrophilicity. This characteristic leads to the easy adhesion of dust, water stains, and grease, resulting in a build-up of dirt over prolonged use. Additionally, the absence of anti-fouling coatings makes it susceptible to contaminant erosion, causing permanent surface damage. Consequently, researchers have developed various surfaces that exhibit superhydrophobic, superhydrophilic, and superamphiphobicity properties to enhance the self-cleaning and abrasion resistance mechanical performance of glass surfaces.^28–30^

#### 2.2.1. Self-cleaning surfaces induced by superhydrophobicity

Superhydrophobic surfaces, with a water contact angle (WCA) ranging from 150° to 180°, were first reported by Ollivier in 1907, who described an almost 180° WCA on a surface composed of soot, rosin powder, and arsenic trioxide.^31^ Since then, research on superhydrophobic surfaces has flourished. Due to their excellent water-repellent properties, when water droplets fall onto these surfaces, they tend to form nearly perfect spheres and then roll off the surface at a slight angle, carrying away contaminants and achieving a self-cleaning effect. In certain instances,^32^ superhydrophobic surfaces can exhibit dynamic behaviors, such as droplets bouncing off the surface upon impact (the Leidenfrost effect), which occurs when the surface temperature is higher than the droplet's boiling point, forming a vapor layer that suspends the droplets.

**Fig. 4:**
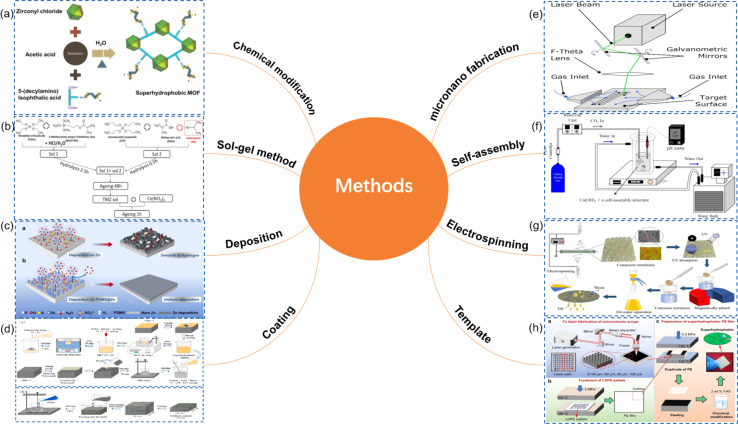
Preparation method of superhydrophobic surface: (a) chemical modification;^33,34^ (b) sol–gel method;^35,36^ (c) deposition;^37,38^ (d) coating;^39–42^ (e) micronano fabrication;^43,44^ (f) self-assembly;^45,46^ (g) electrospinning;^47,48^ (h) template^49,50^

**Fig. 5:**
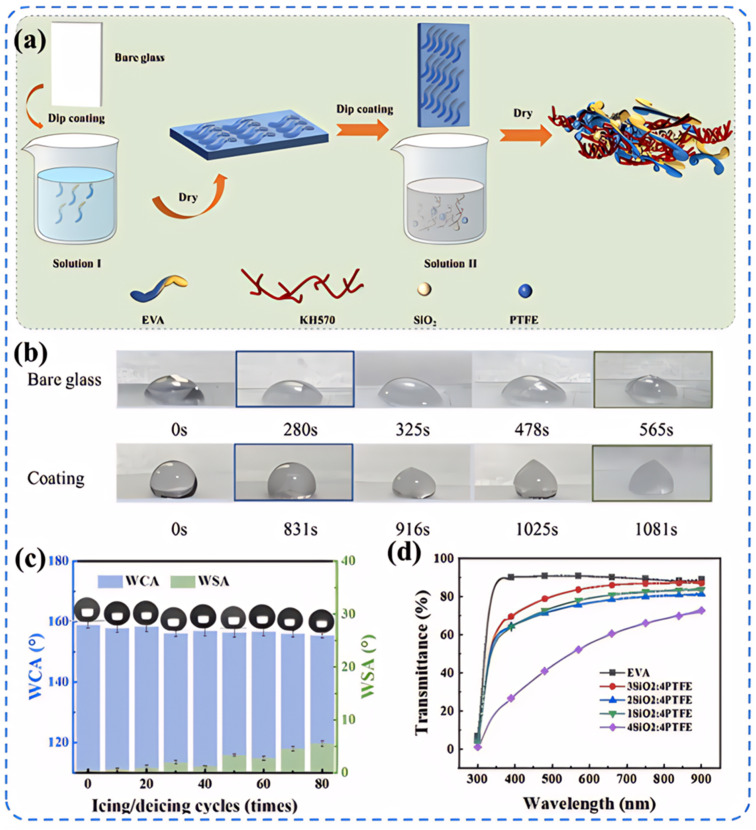
Preparation method of EVA/SiO_2_/KH-570/PTFE coating and its performance characterization: (a) the preparation process flow chart of EVA/SiO_2_/KH-570/PTFE coating; (b) the icing time of smooth glass surfaces and coating surfaces; (c) the effect of the time of icing/decing cycles on the WCA and WSA of EVA/SiO_2_/PTFE/KH-570 coating; (d) UV-vis transmittance curves of EVA/SiO_2_/PTFE/KH-570 coatings with different mass ratios of SiO_2_ to PTFE.^53,54^

Superhydrophobic surfaces typically require a combination of low surface energy materials and specific microstructures to achieve. This combination maximizes the contact angle with water droplets while minimizing the contact area. Fig. 4 illustrates various common methods for preparing superhydrophobic surfaces, each with its characteristics. The roughness and transparency of traditional glass materials often conflict with each other. However, when the microstructure of the glass surface possesses optimal geometric dimensions and shapes, the surface roughness can actually enhance the material's transparency.^51^ Therefore, achieving a superhydrophobic glass surface with high transparency and good abrasion resistance requires a comprehensive consideration of the material's chemical properties and physical structure. In the field of exploring transparent superhydrophobic coatings, Lin *et al.*'s research^52^ presents significant advancements. They utilized femtosecond lasers to create periodic micro-pit structures and self-organized micro–nano features on glass surfaces. By incorporating fluorosilane chemical modification, they developed a superhydrophobic surface that maintains over 90% transparency while enhancing roughness. Liu *et al.*^53^ successfully fabricated an EVA/SiO_2_/PTFE/KH-570 coating through a dip-coating method. This coating not only demonstrated outstanding superhydrophobic performance but also achieved a light transmittance of up to 87%, meeting the strict requirements for transparency in optical applications. Moreover, after enduring durability tests such as sand impact, acid–base solution corrosion, and long-term outdoor exposure, the coating maintained its superhydrophobicity and transparency, showing excellent robustness and chemical stability (Fig. 5).

However, superhydrophobic surfaces may lose their superhydrophobicity when covered by oil stains or other pollutants. When glass surfaces inevitably come into contact with volatile oil vapors from kitchens and hydrocarbons in vehicle exhaust, this can affect their self-cleaning effectiveness.^55,56^

#### 2.2.2. Self-cleaning surfaces induced by superhydrophobicity and photocatalysis

**Fig. 6:**
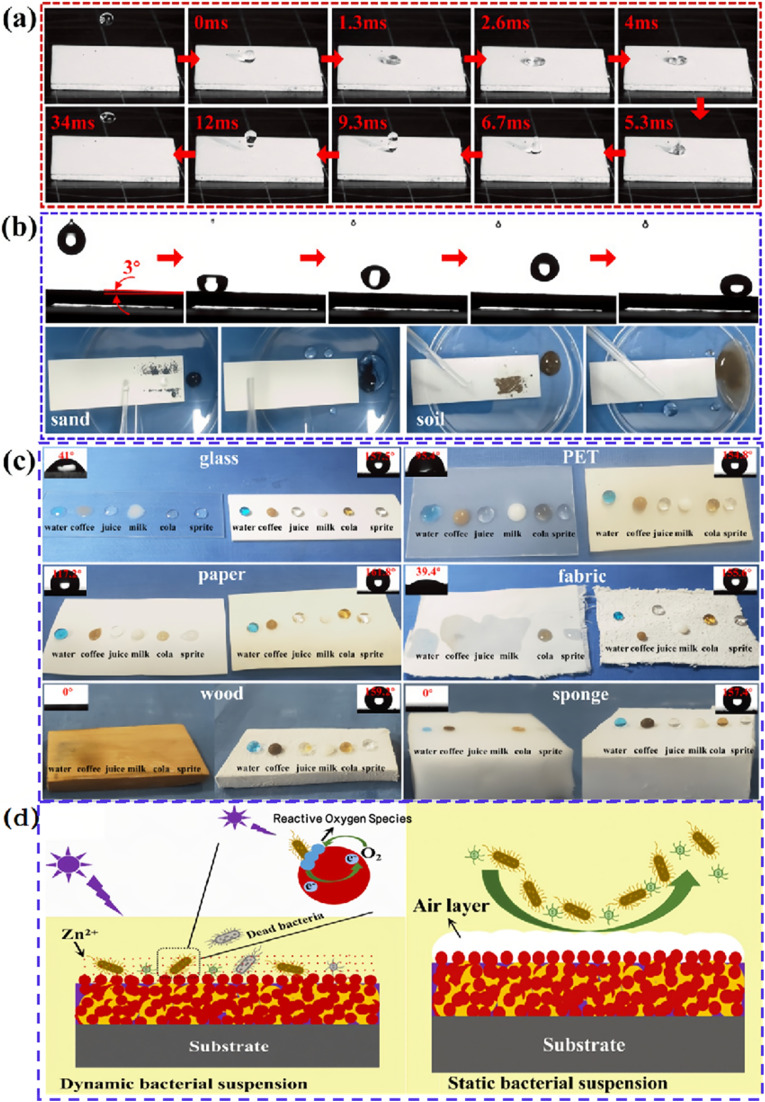
(a) Pictures of 10 μL water droplets impacting/rebounding on the superhydrophobic coating surface; (b) low adhesion property of coating to water droplets and self-cleaning process of quartz sand and soil; (c) liquid repellency of glass, PET film, paper, fabric, wood and sponge sprayed with superhydrophobic coatings to water, coffee, juice, milk, cola and sprite. (d) The mechanism of antibacterial activity in dynamic bacterial suspension and the mechanism of antibacterial adhesion in static bacterial suspension.^57^

The synergistic effect of superhydrophobicity and photocatalytic activity in self-cleaning endows dual-functional surfaces with exceptional long-term self-cleaning capabilities in complex environments. Non-degradable particles on superhydrophobic photocatalytic surfaces can be removed by the rolling of water droplets induced by superhydrophobicity, while degradable pollutants can be catalyzed into harmless gases upon photoexcitation (Fig. 6a).^15^ Therefore, the combination of superhydrophobicity and photocatalysis can exhibit superior performance in the self-cleaning effect on glass surfaces. Cao *et al.*^58^ prepared a multifunctional TiO_2_-based nanocomposite coating by covalently linking TiO_2_ nanoparticles with low surface energy fluorinated silanes. This coating not only demonstrated outstanding superhydrophobic properties but also enhanced photocatalytic and antibacterial characteristics. However, although fluorine-containing coatings generally possess stronger chemical and weather resistance, they can release small amounts of harmful fluoride gases under light exposure. Consequently, further research has been conducted on fluorine-free photocatalytic superhydrophobic coatings.

Ren *et al.*^59^ developed a fluorine-free, robust, and flame-retardant superhydrophobic coating based on a novel branched mercaptan-alkenyl-functionalized siloxane nanocomposite. This coating maintains excellent superhydrophobicity, self-cleaning ability, and durability even under harsh environmental conditions. In another study, Zhao *et al.*^60^ utilized a one-step cold spraying technique to fabricate a fluorine-free superhydrophobic PDMS/STA/TiO_2_ coating, which not only possesses photocatalytic and environmental stability but also exhibits excellent photocatalytic degradation activity against various dyes, along with good chemical, mechanical, and thermal stability. Furthermore, superhydrophobic photocatalytic surfaces have garnered attention for their outstanding antibacterial properties, primarily due to their ability to prevent bacterial aggregation, thus avoiding stains on glass and other surfaces that could further damage the microstructure.^61^ Wu *et al.*^62^ developed a fluorine-free, transparent, anti-reflective photocatalytic superhydrophobic coating that not only offers good self-cleaning functionality but also demonstrates excellent antibacterial capabilities. Zhang *et al.*^57^ created a fluorine-free photocatalytic superhydrophobic paint, capturing with high-speed photography the near-instantaneous rebound of water droplets upon contact with the coating (Fig. 6a). Additionally, Fig. 6b and c display the coating's superhydrophobic performance on various substrates against different liquids, confirming its broad applicability and efficient liquid repellency in practical applications. The study also validated the coating's photocatalytic bactericidal activity in dynamic liquid environments, along with the synergistic effect of its low surface energy and micro–nanostructure trapped air layer in preventing bacterial adhesion, as shown in Fig. 6d. In dynamic suspensions, bacteria experience shear forces due to fluid flow, which may affect their growth rates and metabolic activity. In contrast, bacteria in static suspensions exist in a relatively stable environment, potentially forming biofilms with varying adhesion properties. Moreover, some photocatalytic superhydrophobic surfaces exhibit excellent oil–water separation effects, making them suitable for application on glass surfaces.^63–65^ However, despite the significant effectiveness of photocatalytic technology in removing organic matter, oil pollutants, and bacteria, its efficacy in removing inorganic contaminants, such as dust and mineral deposits, is relatively poor.

The photocatalytic process relies on ample light for efficient energy conversion, but its efficiency can significantly decrease under insufficient lighting conditions, such as overcast days, indoors, or at night. Therefore, developing photocatalytic materials that maintain high activity under low-light conditions is a significant challenge in the current research field. Furthermore, although research combining photocatalytic and superhydrophobic self-cleaning technologies has made certain progress, in-depth studies are still required to ensure the long-term stability and functionality of these technologies in diverse real-world application environments. Future research directions should focus on designing and preparing self-cleaning glass surfaces with higher photocatalytic efficiency, environmental compatibility, and multifunctionality, aiming to meet a variety of application needs and reduce potential negative environmental impacts while achieving cleaning effects.

#### 2.2.3. Self-cleaning surfaces induced by superhydrophilicity

Superhydrophilic surfaces, characterized by extremely low contact angles, nearly 0°, allow water droplets to spread rapidly on the surface, forming a continuous water film. This water film can dissolve dirt and grease on the surface, as superhydrophilic surfaces are typically composed of polar materials that form strong interactions with water molecules. When water flows over a superhydrophilic surface, it carries away the dissolved contaminants, mimicking the effect of rainwater washing leaf surfaces and thus achieving self-cleaning.^9,66^ Ke and Nobi *et al.* have prepared superhydrophilic surfaces on glass and demonstrated the removal of oil contamination and defogging effects through the flow of water films.^67,68^ These studies illustrate the feasibility of inducing self-cleaning effects through surface superhydrophilicity, laying the groundwork for the design and synthesis of superhydrophilic surfaces.

Given the physical properties and low surface roughness of glass, it is less prone to the adhesion and accumulation of contaminants such as dust and water-based pollutants, allowing superhydrophilic surfaces to better exhibit their self-cleaning functions on glass. Although superhydrophilic surfaces may not perform as well as superhydrophobic surfaces in dealing with certain pollutants, such as grease or viscous substances, they still offer significant advantages in other areas.^69^ To enhance the performance of superhydrophilic coatings, it is crucial to ensure their long-term stable adhesion to the glass substrate, which helps prevent delamination or cracking due to insufficient adhesion. Moreover, the self-cleaning ability of superhydrophilic surfaces may be reduced under arid or low-humidity conditions. Inhomogeneity or excessive thickness of the coating can also lead to a decrease in light transmittance, affecting not only the self-cleaning efficiency but also the optical properties of the glass.^4,7,56^

To address these challenges, researchers are actively seeking innovative solutions, including improving coating materials to enhance durability and self-cleaning capabilities, optimizing coating processes to ensure uniformity and appropriate thickness, and developing more enduring and efficient self-cleaning technologies. These efforts aim to enhance the overall performance of superhydrophilic coatings, enabling them to provide excellent self-cleaning functions across a broader range of environmental conditions while maintaining the optical clarity of the glass substrate.

#### 2.2.4. Self-cleaning surfaces induced by superhydrophilicity and photocatalysis

**Fig. 7:**
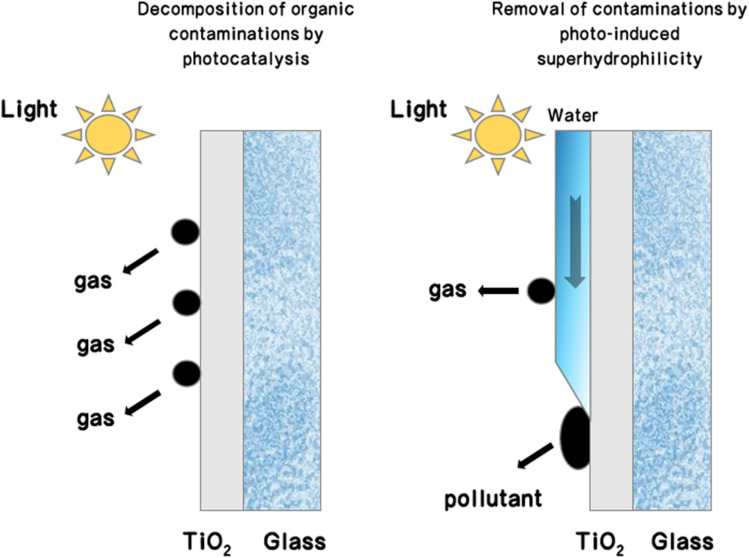
Schematic illustration of the self-cleaning process on TiO_2_ glass surfaces with photocatalytic activity and photo-induced superhydrophilicity.^14,69^

In the research domain of photocatalytic superhydrophilic coatings, the reactive oxygen species generated by photocatalytic action are crucial for the decomposition and oxidation of organic pollutants on the material surface. These pollutants are transformed into water-soluble molecules, while superhydrophilicity ensures that water molecules rapidly spread over the material surface, forming a thin film of water. The function of this water film is not only to help remove soluble substances produced by photocatalytic action but also to further cleanse surface dirt through capillary action, thereby maintaining surface cleanliness.^70,71^ Notably, TiO_2_ and zinc oxide have become two of the earliest materials applied to superhydrophilic surfaces due to their high photocatalytic activity.^72,73^ During the photocatalytic reaction, a large number of hydrophilic hydroxyl groups produced can form hydrogen bonds with water molecules, significantly enhancing the surface's hydrophilicity.^74^ Thus, the combination of TiO_2_ photocatalysis and superhydrophilicity can markedly improve the self-cleaning efficiency of materials.^75,76^Fig. 7 illustrates the self-cleaning process on the TiO_2_ surface, which possesses photocatalytic degradation activity against organic substances and photo-induced superhydrophilic characteristics.

Léonard *et al.*^77^ successfully prepared photocatalytic thin films with a TiO_2_ matrix containing Zn^2+^ and a double-layer structure of TiO_2_/ZnO, observing that the TiO_2_/ZnO double-layer structure samples exhibited exceptional comprehensive performance, including maintaining high hydrophilic properties and good photocatalytic activity. Jrad *et al.*^72^ prepared an In_2_S_3_/br-TiO_2_ heterostructure using a hydrothermal synthesis method, which not only showed excellent photocatalytic activity but also superhydrophilicity, offering potential for its application in water purification. Eshaghi *et al.*^78^ fabricated a TiO_2_–SiO_2_–In_2_O_3_ composite thin film on a glass substrate, which also demonstrated significant photocatalytic activity and superhydrophilicity. Veziroglu *et al.*^79^ prepared a robust film with enhanced photocatalytic and self-cleaning properties by decorating PdO nanoparticles (NPs) on a TiO_2_ structure, showcasing its broad application prospects in outdoor technical surfaces such as photocatalytic and self-cleaning windows. Dundar *et al.*^80^ prepared a TiO_2_ film on a borosilicate glass substrate *via* an ultrasonic spray pyrolysis method and found that the hydrophilicity of the TiO_2_ film deposited at 350 °C was significantly enhanced after ultraviolet treatment, with the WCA value reduced to near 0°, opening up new possibilities for self-cleaning applications. However, it should be noted that such surfaces typically require ultraviolet light or intense sunlight to activate the photocatalytic reaction, which to some extent limits the material's application in environments lacking additional light sources.

In the field of glass applications, superhydrophilic coatings have demonstrated their unique self-cleaning and defogging properties, but their stability and durability in large-scale industrial production and practical applications still face challenges. Particularly on glass surfaces, the adhesion and weather resistance of superhydrophilic coatings need to be further enhanced to withstand adverse weather conditions and long-term ultraviolet exposure. Moreover, the smooth nature of glass surfaces may affect the uniformity and longevity of the coating, thereby impacting its long-term self-cleaning effectiveness. At the same time, the anti-fouling performance of superhydrophilic coatings on glass also needs further optimization to effectively resist the adhesion of various environmental pollutants and grease, ensuring that the clarity and transparency of the glass surface remain unimpaired.

#### 2.2.5. Self-cleaning surface induced by superamphiphobicity

Super amphiphobicity is a unique surface state that combines superhydrophobicity and oleophobicity, characterized by higher roughness and lower surface energy compared to superhydrophobic surfaces.^81,82^ The increased roughness of the surface leads to the formation of more micro protrusions and depressions, which enhance the air cushioning effect between the liquid and the solid surface. When a liquid droplet comes into contact with such a surface, air becomes trapped within the microstructures, creating a cushion that further reduces the contact area between the liquid and the solid surface. The lower surface energy implies weaker intermolecular forces, which helps to minimize the adsorption of the liquid to the solid surface.^83^ On super amphiphobic surfaces with lower surface energy, liquid molecules tend to maintain their shape rather than being adsorbed by the solid surface, thereby enhancing the oleophobic properties. Concurrently, as the liquid rolls off the surface, it removes any contaminants attached to it, achieving self-cleaning action induced by superhydrophobicity.

**Fig. 8:**
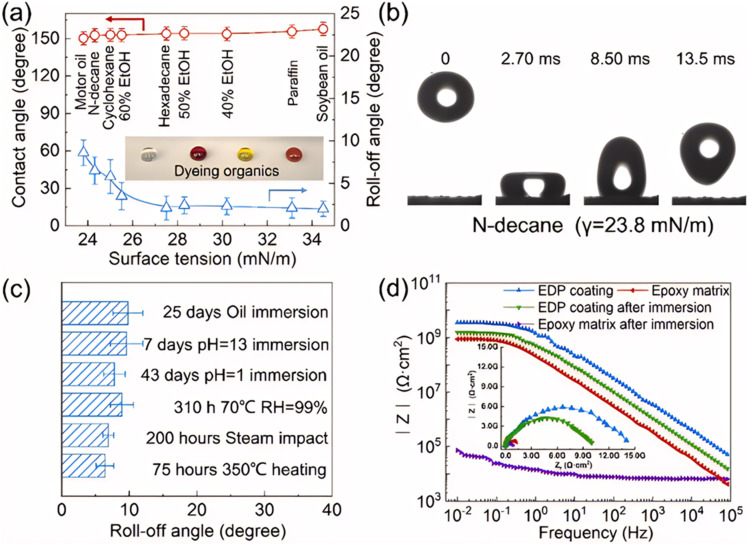
Super liquid-repelling and comprehensive protection properties for supermaphiphobic EPD coating. (a) The contact angles and roll-off angles of liquids with various surface tension on the coating. (b) Typical time-lapse images of the impacting tests on the coating. (c) Comprehensive stability of the coating after various environment erosion. (d) Bode modulus of the coatings before and after the immersion for different time, inserting the Nyquist plot. Epoxy coating immersed for 15 days, while EPD coating immersed over 70 days.^85^

Ren *et al.*^84^ successfully fabricated a super amphiphobic coating with high thermal conductivity, excellent wear resistance, and enduring corrosion resistance by combining hierarchical filling of modified flaky graphite and modified Al_2_O_3_ through a two-step spraying process. Xuan *et al.*,^85^ on the other hand, prepared a super amphiphobic coating using a combination of layer-by-layer self-assembly and electrophoretic deposition (EPD) techniques. This coating not only demonstrated exceptional corrosion resistance but also maintained its super amphiphobic properties under various chemically aggressive conditions, such as oil immersion and acid–base erosion. Fig. 8 illustrates the coating's excellent superhydrophobicity and comprehensive protective performance, indicating its potential application on glass surfaces.

**Fig. 9:**
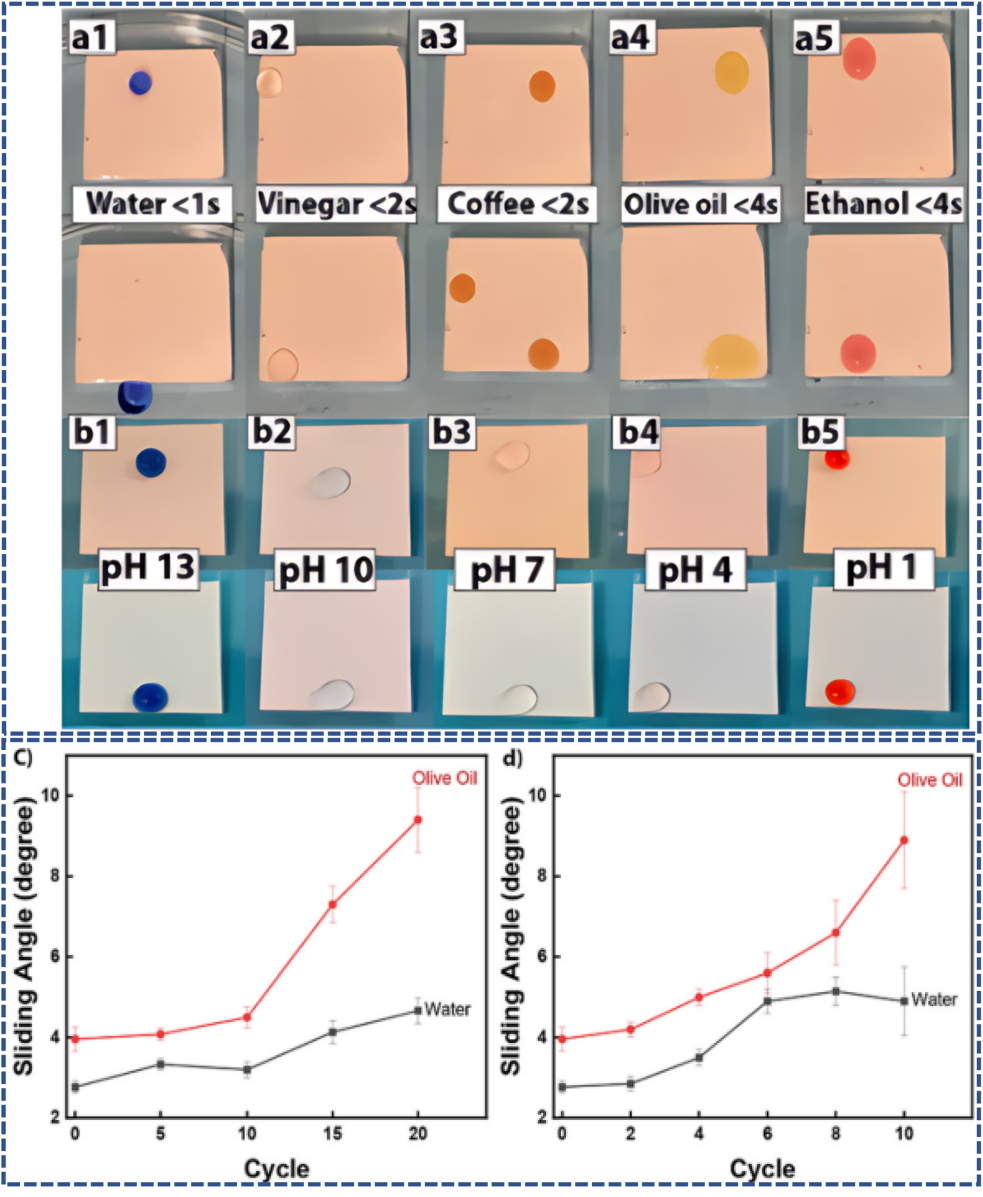
(a1–a5) Repellency of various liquids tested at 14° angle. (b1–b5) Repellency of water at different pH indicates the immiscibility of lubrication and the working liquid. (c) SA of the coating 20 cycles of heating at 120 °C and cooling to −18 °C and (d) SA of the coating after 10 cycles (0.2 ml min^−1^, 10 min per cycle) of water flow test.^86^

On the other hand, Amir *et al.*^86^ utilized polydimethylsiloxane (PDMS) as a substrate and successfully created a super amphiphobic coating by chemically bonding and physically capturing ZnO particles and hydrophobically treated TiO_2_ nanoparticles. As shown in Fig. 9, the coating exhibited remarkable omniphobicity and immiscibility. After rigorous mechanical, thermal stability, and chemical pH value tests, the coating's hydrophobicity and oleophobicity were preserved, demonstrating the significant potential of this simple and cost-effective coating for practical applications. It offers new solutions for various scenarios, including self-cleaning glass and anti-icing measures.

Regrettably, these surfaces often rely on complex micro–nano structures to achieve their properties, which can degrade under mechanical damage, wear, and environmental corrosion, leading to a decline in self-cleaning effectiveness. Additionally, the fabrication of these surfaces typically requires costly materials and intricate processes, increasing production costs and limiting their application in large-scale industrialization. Furthermore, super amphiphobic coatings may lose stability in extreme environments, such as high temperatures and strong acids or bases, and may not maintain their functionality over extended periods.

#### 2.2.6. Summary of glass self-cleaning surface types

The application of self-cleaning glass surfaces is summarized in Table 1. Superhydrophobicity and superamphiphobicity rely on their unique wetting characteristics to prevent the adhesion of water-based and oil-based pollutants, significantly enhancing the self-cleaning performance of the surface. Photocatalysis, often combined with superhydrophilicity, can effectively decompose and remove organic pollutants by exciting photosensitive materials such as TiO_2_, achieving the self-cleaning effect of the surface.^104,105^ The characteristics of these self-cleaning surfaces provide a theoretical foundation and practical guidance for the research and application of self-cleaning glass technologies.

**Table 1:** The type of self-cleaning surfaces applied on the glass surface

Typical properties for self-cleaning effect	Application methods	Inducing material	Target pollutants for self-cleaning effect	Transparency	Ref.
Superhydrophobicity, amphiphobicity	Coated on glass	PDMS, M-BPE, SiO_2_ nanoparticles	Oil, milk, cola, juice, orange, tea, ink	Opaque	87
Superhydrophobiciy	Nanopreparation	Hydrogen fluoride gas	Muddy water and soil dust	Excellent transparency	88
Superhydrophobiciy	Hot embossing	SiO_2_ nanoparticles	Muddy water and soil dust	Partial transparency	89
Superhydrophobiciy	Nanosecond laser ablation and heat treatment	Zr-based metallic glass surface	Muddy wate and water contamination	Opaque	90
Superhydrophobicity, amphiphobicity	Laser ablation and chemical modification	Fluorosilane modification	Dust, oil	Excellent transparency	91
Photocatalysis, superhydrophobicity	Coated on glass	PDMS/STA/TiO_2_	MB (Methylene blue), ink, graphite powder	Excellent transparency	60
Photocatalysis	Melt quenching technique	Bi_2_O_3_, P_2_O_5_, TeO_2_	Rhodamine B	Excellent transparency	92
Photocatalysis, superhydrophilicity	Sol–gel dip coating process	TiO_2_–SiO_2_–In_2_O_3_	Water-based pollutant	Partial transparency	78
Photocatalysis	Coated on glass	PdO, TiO_2_	MB, Oleic acid	Opaque	79
Superhydrophilicity	Coated on glass	TiO_2_–SiO_2_-PAA	MB, dust	Excellent transparency	93
Superamphiphobicity	Coated on glass	EP/PTFE/FG/Al2O3	Glycerin, ethylene glycol	Opaque	84
Superamphiphobicity	Femtosecond laser induced printing	PTEF	Water-based pollutant, oil	Partial transparency	94
Photocatalysis, superhydrophilicity	Deposited on glass	TiO_2_/WO_3_	Carbon accumulation, Dust, oil	Excellent transparency	95
Superhydrophilicity	Coated on glass	TiO_2_-KH550-PEG	MB, dust	Excellent transparency	96
Superhydrophobicity	Deposited on glass	SiO_2_, FAS	Water-based pollutant	Excellent transparency	53
Photocatalysis	Deposited on glass	ZnO/CuO	Organic pollutants in water, acriflavine	Opaque	97
Photocatalysis	Coated on glass	Ag/In_2_O_3_	Sulfisoxazole, tetracycline hydrochloride, rhodamine B	Opaque	98
Superhydrophobicity	Hydrothermal method	RGO/Er_2_O_3_	Dust	Excellent transparency	99
Photocatalysis, superhydrophilicity	Coated on glass	Quantum-size TiO_2_	MB, 4-chlorophenol	Excellent transparency	100
Superhydrophilicity	One-time firing route	Titanic-based-crystallites	Ink, miso sauce	Opaque	101
Photocatalysis, superhydrophilicity	Coated on glass	PVDF-TiO_2_ -HAP	MB, MO	Opaque	102
Superhydrophilicity	Coated on glass	SiO_2_, Al_2_O_3_, AOS, TEOS	Oil, dust, bird droppings	Excellent transparency	103

However, there is currently a lack of uniform evaluation standards for self-cleaning surfaces, and a clear assessment system to measure the performance of different self-cleaning surfaces is missing. This is crucial for the selection and application of materials. Existing evaluation methods largely depend on laboratory tests, such as contact angle measurement, sliding angle testing, and photocatalytic activity evaluation, which may not fully reflect the performance of materials in real-world applications.

## 3 The durability of the glass self-cleaning surface

Considering the application environment of glass, self-cleaning surfaces exhibit poor durability due to the influence of various factors. For instance, the service life of certain special coatings or materials may be diminished by ultraviolet radiation, chemical corrosion, or physical impact leading to wear;^57,106^ the natural aging of the materials, which gradually lose their original properties over time; and the friction during cleaning processes and the use of chemical cleaning agents, all of which can accelerate the degradation of self-cleaning surfaces.^107,108^ Therefore, when selecting self-cleaning surfaces for different application environments, their durability should be comprehensively considered.

### 3.1. Evaluation method for durability of glass self-cleaning surface

**Fig. 10:**
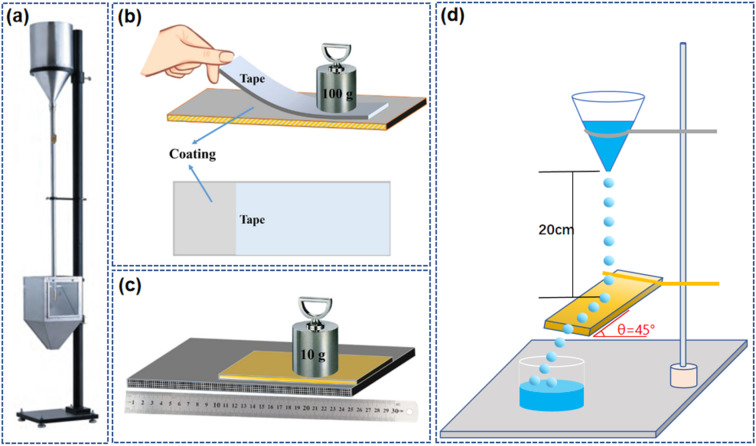
Test method for robustness of self-cleaning coating: (a) sand falling test.^114,115^ Tape peeling test (b) and sandpaper abrasion test(c).^111^ (d) Water-impacting test.^116^

When applying coatings on glass, the mechanical toughness of the coating is of utmost importance. It determines the coating's resistance to impacts, abrasion, or temperature changes. Coatings with good mechanical toughness can better absorb and disperse these external forces, preventing cracks or delamination, thereby extending the service life of the coating and maintaining its functionality. Damage to micro–nano structures or chemical composition can negatively impact the performance of the coating, greatly limiting its application.^109,110^ Generally, tests to assess the robustness of self-cleaning coatings include sand impact, tape peeling, sandpaper abrasion, and water impact tests,^111–116^ as shown in Fig. 10.

The sand impact test is used to evaluate the resistance of a coating or other material surfaces to abrasion. In this test, abrasive particles (usually sand) are dropped through a guide tube and strike the test specimen until the substrate is exposed. Abrasion resistance is represented by the volume of abrasives, that is, the amount of abrasives required to wear through a certain thickness of the coating.^56,117^ Tape peeling is a test method to assess the adhesion strength between a coating or surface treatment layer and the substrate. 3M tape is applied to the coating surface and then quickly stripped off at a certain angle and speed, simulating the delamination or shedding that the coating may encounter during actual use. The adhesion strength and durability of the coating can be evaluated by measuring the force required to strip the tape.^111^ Sandpaper abrasion involves physically scraping the coating surface with abrasive particles of sandpaper, simulating the damage that hard particles or rough objects may cause when in contact with the coating. This type of abrasion test helps to understand the coating's ability to resist daily friction and wear in practical applications.^111,118^ The water impact test simulates the natural impact of rainwater or other water sources on the coating surface to examine the self-cleaning effect and durability of the coating in actual environments. By observing the contact angle, sliding angle, and rolling behavior of water droplets on the coating surface, the hydrophilic or hydrophobic characteristics of the coating are evaluated, and it is examined whether the coating can remove surface contaminants through the flow of water.^113^

Commonly used test methods for the chemical stability of self-cleaning surfaces currently include acid and alkali resistance tests, salt spray tests, solvent immersion tests, humidity and heat aging tests, and ultraviolet (UV) aging tests. The specific testing methods are as follows:

Acid, alkali, and salt tests: the self-cleaning surface is exposed to acidic or alkaline solutions with different pH values to observe whether the superhydrophobic properties of the surface change after a certain period, and whether there is any corrosion or delamination.^56,110^

Salt spray test: the self-cleaning surface is placed in a high-concentration salt solution to simulate the corrosive effects in marine or high-salt environments, assessing the coating's corrosion resistance.^111,112^

Solvent immersion test: the surface is immersed in various organic solvents, such as alcohol, gasoline, acetone, *etc.*, to test the surface's tolerance to common solvents.^106,108^

Humidity and heat aging test: the self-cleaning surface is subjected to accelerated aging in a high-temperature and high-humidity environment to simulate the environmental conditions that may be encountered during long-term outdoor use.^113,114^

UV aging test: the self-cleaning surface is exposed to ultraviolet light sources to evaluate its stability under long-term sunlight conditions.^117–119^

### 3.2. Robustness of the glass self-cleaning surface

The durability of self-cleaning surfaces refers to the ability of these surfaces to maintain their self-cleaning capabilities while withstanding the long-term effects of environmental factors such as ultraviolet radiation, mechanical wear, and chemical corrosion without performance degradation. When applying self-cleaning coatings on glass surfaces, such mechanical damage is inevitable.^57,119^Table 2 shows the robustness of self-cleaning surfaces in recent studies. It can be seen that self-cleaning surfaces still exhibit a certain degree of robustness under the actions of sand falling, sandpaper abrasion, and water impact.

**Table 2:** Robustness of self-cleaning surface in recent research

Types self-cleaning surface	Preparation	Test methods	Test results	Ref.
Superhydrophilicity	Gafting polymerization on silanized glass	Linear reciprocating abrasion	WCA ≈ 10°	119
Superhydrophilicity	Sprayed ZrO_2_/TiO_2_/HSN on glass	Sandpaper abrasion	WCA > 150°	120
Superhydrophobicity	Sprayed hydrophobic adhesive and SiO_2_ nanoparticles on substrate	Sand falling, water-impacting	WCA ≈ 152°	121
Superhydrophobicity	Electrophoretic deposition and self-assembly of fluorinated nanoparticles on a pre-roughened polymer layer	Manual grinding, friction test	WCA > 150°	85
Superhydrophiliciy	Dip-coating glass in a solution of nanoparticles and binders, then annealing	Grinding abrasion	After 500 times WCA > 10°	103
Superhydrophobicity	Sprayed modified nanoparticles suspension on adhesive-coated substrate	sandpaper abrasion, sand falling, scraping test	WCA > 135°	122
Superhydrophilicity	Synthesized PVA/GO@MOF membrane *via* chemical crosslinking	sandpaper abrasion	OCA > 150°	123
Superhydrophobicity	Coating glass with a solution derived from TEOS and Me-MQ, then annealing	Sand falling sandpaper abrasion	WCA > 127.5°	62
Photocatalysis, hydrophilicity	Dip-coating with SSNs, P25 and ACSS mixtures	Pencil scratching, tape peeling	Stable self-cleaning effect	124

Enhancing the robustness of self-cleaning surfaces is closely related to the adhesive materials, surface morphology, and structure. For instance, Li *et al.*^125^ prepared a robust ultraviolet-resistant hierarchically textured superhydrophobic surface based on a water glass interface enhancement, where the water glass strengthened the interfacial force between inorganic SiO_2_ and ZnO particles and the substrate, demonstrating excellent robustness. For glass, it is essential to have good light transmittance while ensuring durability. Ke *et al.*^67^ prepared an oxide-based coating with micro–nano roughness, which exhibited high transparency and good mechanical strength. The morphology and microstructure of the coating, by creating a micro–nano hierarchical surface roughness combined with the modification of low surface energy materials, achieved super-wettability on glass surfaces. However, in practical applications, coatings often undergo the combined effects of multiple environmental factors, and it is still challenging to establish an equivalent evaluation mechanism compared to indoor wear and impact tests. Therefore, to ensure that the performance of the coating in actual applications matches the laboratory results, long-term field testing and monitoring are required, and it is considered necessary to adjust the laboratory test conditions to more closely resemble the actual usage environment.

### 3.3. Chemical stability of the glass self-cleaning surface

The chemical stability of self-cleaning surfaces refers to the ability of coatings or surface materials to maintain their physical and chemical properties without significant changes when exposed to various chemical substances such as acids, bases, salts, and organic solvents. Super-wetting surfaces, especially superhydrophobic surfaces, may have relatively poor chemical stability because the coatings or modification layers that constitute these surfaces often have higher chemical activity or are made of materials susceptible to chemical erosion. For instance, some superhydrophobic coatings may contain organic components that are easily decomposed by ultraviolet light or rely on specific chemical functional groups to achieve their unique wetting properties. These functional groups may undergo degradation, oxidation, or other chemical reactions upon contact with certain chemicals or prolonged exposure to harsh environmental conditions, leading to a decline or failure of surface performance.^15,60^ Furthermore, the microstructure of super-wetting surfaces can be quite complex, and any damage to these structures can affect their chemical stability. Currently, chemical stability is primarily evaluated through acid–base-salt immersion and UV aging tests (Table 3).

**Table 3:** Chemical stability of self-cleaning surface in recent research

Types self-cleaning surface	Preparation	Test methods	Test results	Ref.
Superhydrophobicity	Sprayed hydrophobic adhesive and SiO_2_ nanoparticles on substrate	NaCl, different pH solution immersion	WCA > 155°	121
Superhydrophobicity	Electrophoretic deposition and self-assembly of fluorinated nanoparticles on a pre-roughened polymer layer	NaCl, acid, alkali and oil immersion	WCA > 150°	85
			WRA < 10°	
Superhydrophilicity	Dip-coating glass in a solution of nanoparticles and binders, then annealing	Boiling water	After 35 min WCA > 10°	103
Superhydrophobicity	Sprayed modified nanoparticles suspension on adhesive-coated substrate	UV aging	WCA > 150°	122
Superhydrophilicity	Synthesized PVA/GO@MOF membrane *via* chemical crosslinking	NaCl, acid, alkali	OCA > 150°	123
Superhydrophobicity	Coating glass with a solution derived from TEOS and Me-MQ, then annealing	Extreme temperature test, UV aging	WCA > 137°	103

Self-cleaning glass is often subject to corrosion from domestic sewage and rainwater, making its resistance to various aqueous solutions crucial. Acidic or alkaline environments may cause chemical erosion or hydrolysis of the coating, leading to the destruction of surface functional groups or the disintegration of the coating structure.^123,126^ Salts can affect the surface roughness and chemical composition of the coating through salting-out effects or crystal deposition, thereby weakening the coating's adhesion and stability.^117,127^ Additionally, long-term exposure to saline environments can accelerate the corrosion process of the coating, especially in marine or high-salt fog areas. Prolonged UV radiation can excite photosensitive materials in the coating, such as certain organic groups or metal oxides, leading to photochemical reactions. These reactions may cause the deterioration of the material's surface structure, including the breaking of chemical bonds, loss of functional groups, or phase changes.^125,128,129^

Self-cleaning coatings use polymeric materials as the matrix, with a closely arranged molecular structure that reduces pores and defects, thereby decreasing the likelihood of acid–base ion penetration. In acid–base resistance tests, hydrogen ions in acidic solutions may react with hydrophilic groups in the coating, while the corrosion from strongly alkaline solutions may cause some polymer coatings to lose surface properties.^126^ Therefore, when applying self-cleaning coatings on glass, the physical and chemical characteristics of the coating and the actual usage scenarios of the glass should be considered. Moreover, for photocatalytic-induced self-cleaning surfaces, specific stabilizers and antioxidants are added to the coating to enhance its corrosion resistance.^95,130^

In summary, the research on the durability of self-cleaning surfaces has achieved significant results at the laboratory level. However, despite the existence of tests such as sand impact, tape peeling, sandpaper abrasion, and water impact to assess the robustness of coatings, coatings in practical applications are often subject to the combined effects of multiple environmental factors, and it is currently challenging to establish an equivalent evaluation mechanism compared to indoor wear and impact tests. In terms of chemical stability, there are various standards and methods for corrosion resistance testing. To ensure the accuracy and practicality of test results, it is recommended to adjust the test conditions in the laboratory appropriately after long-term field application tests and continuous monitoring, making them closer to the actual application environment. Additionally, establishing a unified evaluation system will help standardize the chemical stability testing process, improving the comparability and reliability of test results.

## 4 Application of self-cleaning glass

In recent years, a multitude of self-cleaning glass materials have been developed and successfully applied across various fields. These self-cleaning glasses not only exhibit excellent anti-pollution and self-cleaning capabilities but also play significant roles in antibacterial properties, ultraviolet resistance, corrosion resistance, frost prevention, defogging, and oil–water separation.^69,119,131^ The broadness of these applications is attributed to the unique performance of photocatalytic materials, as well as the superhydrophilic and superhydrophobic characteristics of self-cleaning surfaces. However, not all features of self-cleaning surfaces can be effectively utilized. For instance, some photocatalytic materials may require specific wavelengths of light to become activated, and in certain scenarios, due to limitations in lighting conditions, the self-cleaning effects of these materials may not be optimal. Therefore, despite the promising prospects for the application of self-cleaning glass, it is essential to consider the compatibility of environmental factors and material characteristics in practical applications.

### 4.1. Anti-bacteria and anti-microbial properties

The application prospects of antibacterial properties are vast in food packaging, medical devices, transportation equipment, and building materials.^132^ Ordinary glass, under the long-term erosion of bacteria, leaves stubborn stains on its surface and affects the microstructure, leading to a decrease in durability. Therefore, when designing self-cleaning glass, its antibacterial and antimicrobial characteristics should also be considered comprehensively.

The realization of antibacterial and antimicrobial properties is typically achieved by integrating photocatalytic nanoparticles (such as TiO_2_) on the material surface, which generate strong oxidative free radicals under light exposure to destroy bacterial and microbial cells.^133,134^ Meanwhile, the surface roughness, chemical composition, and charge state of the material can affect the adhesion of microorganisms.^135,136^ For instance, the excellent water-repellency of superhydrophobic and superamphiphobic surfaces allows water droplets to roll over the surface, carrying away attached bacteria and microorganisms. Superhydrophilic surfaces, in conjunction with photocatalysis, reduce bacterial adhesion by forming a hydration layer and, combined with the highly active free radicals produced by photocatalytic materials under visible light, such as hydroxyl radicals and superoxide radicals, can destroy bacterial cell structures, leading to bacterial death and achieving antibacterial effects.^137,138^ Furthermore, by doping or coating antimicrobial agents like silver, copper, or specific polymers directly onto the material surface, substances that inhibit or kill microorganisms can be released.^134^

**Fig. 11:**
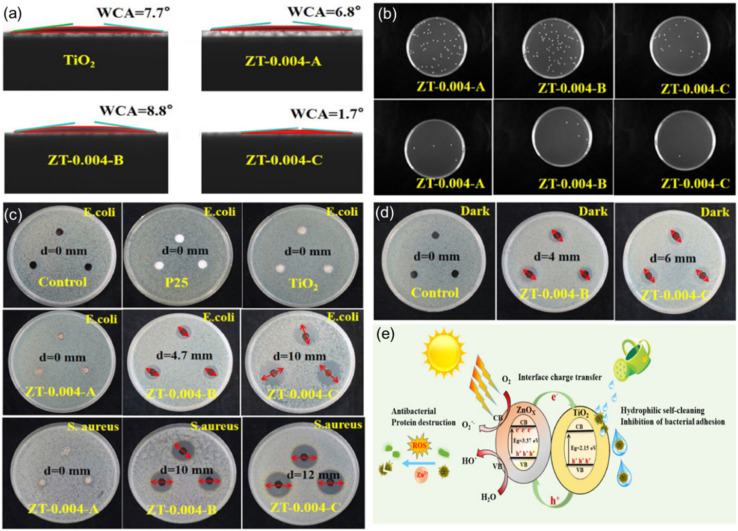
(a) Water contact angle of TiO_2_ and different ZnOX/TiO_2_ films. (b) Number of colonies of *E. coli* adhering to ZnOX/TiO_2_ films, the dilution factor differs by an order of magnitude between the upper and lower figures. (c) Diffusion test against *E. coli* under visible light irradiation and diffusion test against *S. aureus* under visible light irradiation. (d) Diffusion test on *E. coli* in the dark. (e) Schematic diagram of the antibacterial self-cleaning mechanism of ZnOX/TiO_2_.^139^

Thus, self-cleaning surfaces that combine photocatalysis and super-wetting characteristics not only provide immediate antibacterial action but also enhance the surface's long-term cleanliness and hygiene maintenance by reducing microbial adhesion and promoting their removal. This feature enables it to adapt to a variety of different usage environments. In the research by Zhou *et al.*,^139^ superhydrophilic ZnOX/TiO_2_ nanocomposites were prepared, successfully demonstrating their self-cleaning and antibacterial properties under visible light exposure. As shown in Fig. 11b and c further confirm the ability of these materials to inhibit bacterial adhesion and to inactivate *Escherichia coli* and *Staphylococcus aureus* under visible light. Notably, the ZT-0.004-C sample exhibited the largest inhibition zones against *E. coli* and *S. aureus* under visible light, reaching 10 mm and 12 mm, respectively (Fig. 11c). Moreover, Fig. 11d shows that the ZT-0.004-C sample can effectively inactivate *E. coli* even in the absence of light. These findings reveal the potential application prospects of ZnOX/TiO_2_ nanocomposites in the coating of medical facilities and public space surfaces. The antibacterial self-cleaning mechanism illustrated in Fig. 11e further explains how the material achieves antibacterial effects by generating reactive oxygen species and releasing Zn^2+^ ions under light exposure.

### 4.2. Anti-UV and heat reflection

UV-resistant glass is widely used in residential buildings, automobiles, museums, and any settings where protection against UV damage is required. UV can accelerate the aging process of materials, leading to color fading, degradation of material properties, and potential chemical structural changes. For humans, prolonged exposure to UV can result in skin damage or even skin cancer.^140,141^

The UV resistance of glass is achieved by incorporating specific chemical substances during its manufacturing process, such as cobalt oxide, nickel oxide, or rare earth elements, which absorb or reflect ultraviolet rays, thereby reducing the amount of UV radiation that penetrates the glass. For instance, some chemical substances like UV absorbers can transform UV into lower-energy heat, while physical barriers such as TiO_2_ nanoparticles can scatter UV, reducing their direct exposure to surfaces.^142,143^ Additionally, special surface designs, like micro–nano structures, can scatter or absorb UV through the geometric characteristics of the structure, achieving UV resistance.^144,145^

**Fig. 12:**
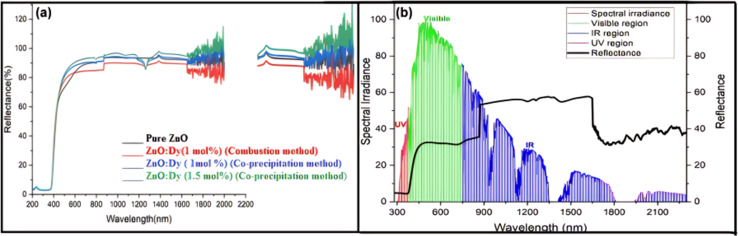
(a) Diffuse reflectance spectra for zinc oxide pigment. (b) Diffused reflectance spectra for glass plate coated using ZnO:Dy pigment.^148^

Concurrently, UV resistance and heat reflection functions often appear together. Heat reflection is achieved by applying a special low-emissivity coating on the glass surface, which reflects the infrared portion of solar radiation and reduces heat transfer.^146,147^ Factors affecting heat reflection include the material's infrared reflectance, the crystal structure and phase purity of the pigments, the surface morphology and particle size of the pigments, the dispersibility and compatibility of the pigments in the coating, and the thickness and uniformity of the coating. These factors collectively determine the material's ability to absorb and reflect light in the infrared region of the solar spectrum. Pawade *et al.*^148^ have extensively discussed the infrared reflectance properties of ZnO:Dy^3+^ pigments on glass surfaces. Specifically, Fig. 12a illustrates the diffuse reflectance spectrum of the ZnO pigment, revealing its spectral reflectance characteristics in the range of 200 nm to 2200 nm, with a strong reflectance observed in the 1300–2100 nm range, indicating the pigment's excellent near-infrared reflectance capability. Furthermore, Fig. 12b shows the diffuse reflectance spectrum of the heat-reflective coating prepared with ZnO:Dy pigment on a glass plate, which, when compared with the solar spectral irradiance curve, demonstrates a good reflectance of 40–60%, further confirming the pigment's potential in reducing surface thermal effects. In coating design, UV-resistant and heat-reflective materials can be combined in a single coating, with the self-cleaning coating as the outermost layer, ensuring the chemical stability and physical performance of each material layer.

### 4.3. Anti-fog, anti-freeze, anti-corrosion

The fogging phenomenon on automotive windows and building glass surfaces caused by temperature differences has long been a concern and a frequent subject of criticism from the public. The defogging performance of self-cleaning glass is primarily attributed to its superhydrophobic or superhydrophilic surface characteristics. Superhydrophobic surfaces can inhibit the condensation of water droplets, maintaining the transparency of the glass. In contrast, superhydrophilic surfaces facilitate the spread of water droplets; when water vapor encounters such surfaces, it rapidly condenses into a thin film, reducing light scattering and thus preserving transparency and preventing fog formation.^149,150^ Superhydrophilic surface coatings are also effective in preventing fog formation on medical equipment, where it is essential to ensure the coatings' biocompatibility, non-toxicity, and resistance to common sterilization methods such as high-temperature steam and chemical disinfection. Additionally, the coatings must possess excellent durability to withstand the frequent use and cleaning demands of medical devices.^129,151^ Therefore, self-cleaning surfaces hold great promise in the field of anti-fogging applications.

**Fig. 13:**
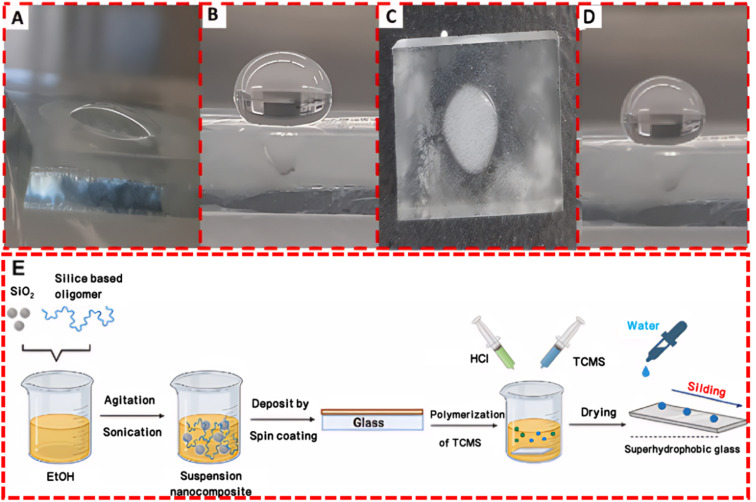
Anti-icing test: (A) bare surface at *t*_0_, (B) coated surface at *t*_0_, (C) bare surface after 20 min at −10 °C, (D) coated surface after 20 min at −10 °C. (E) Schematic illustration of the preparation of the transparent superhydrophobic coating.^153^

The formation of ice layers on glass surfaces reduces light transmittance, increases structural load, and causes surface damage. The frost prevention mechanism of self-cleaning glass surfaces is mainly based on superhydrophobic properties. The rough surface formed by micro–nano structures reduces the contact area between water droplets and the coating, thereby decreasing heat conduction efficiency. At the same time, it increases the contact area between water droplets and air, enhancing heat acquisition through radiation and convection. This results in reduced heat loss from water droplets on superhydrophobic surfaces, and the altered heat balance leads to a longer time required for water droplets to freeze on these surfaces.^83,152^ Nomeir *et al.*^153^ developed a durable and transparent superhydrophobic coating with temperature-controlled multi-scale roughness, which, through the combination of specific nano-fibers and larger-sized nanoparticles, achieved excellent superhydrophobicity, transparency, and durability, demonstrating significant anti-freezing performance by markedly delaying the freezing process of water droplets even at −10 °C (Fig. 13). Superhydrophilic coatings, however, are generally not suitable for frost prevention as they attract water molecules and promote their spread and freezing on the surface. Therefore, for frost prevention applications, the design of self-cleaning glass should consider superhydrophobic surfaces.

**Fig. 14:**
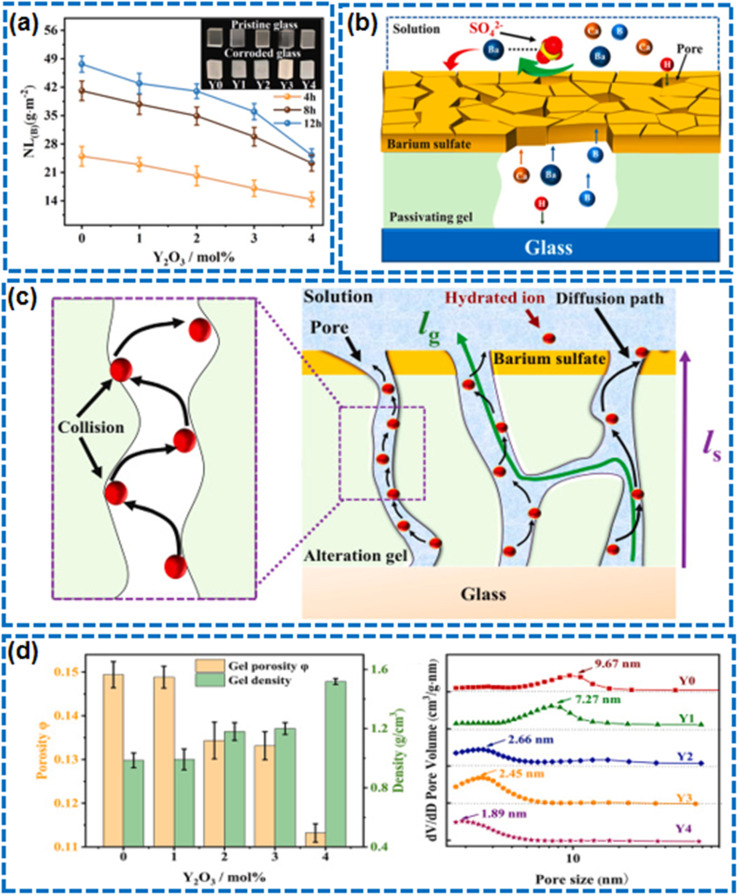
(a) The effect of Y_2_O_3_ doping on acid resistance of the glass. (b) Schematic diagram of ion diffusion in passivating gel. (c) Schematic diagram of hydrated ion diffusion in passivating gel. (d) Density and porosity of alteration products and Pore size distribution of the alteration products.^158^

Corrosion of self-cleaning glass is a primary cause affecting transparency and durability.^154,155^ Glass corrosion is typically caused by chemical reactions between the glass and substances in acidic, alkaline, or saline environments. These reactions can lead to the deconstruction of the glass network structure, especially when the silicate network in the glass is attacked by protons such as H_3_O^+^, promoting the formation of non-bridging oxygen atoms in the network, increasing its solubility, and causing corrosion. Moreover, moisture and temperature fluctuations in the environment can accelerate the corrosion process, as they facilitate the migration of ions and the penetration of reactants, leading to the formation of a porous gel layer on the glass surface that further exacerbates corrosion.^90,156–158^ In this process, Li *et al.*'s research showed that yttrium oxide doping affects the acid resistance of borosilicate glass, finding that a passivating gel layer with smaller pore diameter, higher tortuosity, and lower porosity formed during the corrosion process effectively inhibits ion migration within the gel layer^158^ (Fig. 14). It is noteworthy that self-cleaning surfaces induced by superhydrophobicity, with their excellent water-repellent properties, can effectively block the penetration of aqueous solutions onto the glass surface. However, for self-cleaning surfaces induced by photocatalysis and superhydrophilicity, their resistance to corrosion from acid, base, and salt solutions is relatively weaker.^56,159^ Therefore, when designing the self-cleaning surfaces of glass, this corrosion-resistant characteristic should be carefully considered.

## 5 Conclusions and outlook

This review summarizes the various applications of self-cleaning glass surface technology and its durability issues, and discusses the applicability and limitations of different types of self-cleaning surfaces. By analyzing the strengths and weaknesses of these surfaces in practical applications, we can see the significant progress in self-cleaning surface technology, as well as the limitations encountered in real-world use.

Superhydrophobic surfaces are often vulnerable to mechanical damage and chemical corrosion in long-term use due to the complexity of their surface micro–nano structures, which limits their durability to a certain extent. In contrast, the photocatalytic surface has good chemical stability and can resist the erosion of the external environment to a certain extent, but its dependence on light intensity and the weakening of photocatalytic activity by pollutant accumulation are also a major challenge. Other types of self-cleaning coatings, such as super-hydrophilic coatings, perform well in preventing liquid contamination, but their ability to resist contamination is often limited in high humidity environments. In addition, the existing coating preparation process is complex and costly, which limits the possibility of large-scale industrial application.

To address these issues, future research needs to further tackle the following challenges:

(1) Current superhydrophobic and superhydrophilic surfaces are easily affected by the external environment in practical applications, leading to a decline in their self-cleaning performance. Therefore, future research should focus on developing more wear-resistant and stable materials, while optimizing the surface micro–nano structures to enhance their impact resistance and corrosion resistance.

(2) A major obstacle to the existing self-cleaning surface technology is its high preparation cost, which hinders widespread application. Future research should prioritize the development of efficient, low-cost preparation processes to achieve the feasibility of large-scale application.

(3) With the continuous development of self-cleaning surface technology, how to endow these surfaces with additional functions (such as antibacterial, corrosion resistance, anti-icing, *etc.*) will become an important direction for future research. Multifunctional coatings will further expand their application scenarios, especially in harsh environments.

(4) Currently, there is a lack of uniform assessment standards for self-cleaning surfaces, and there is a significant variation in evaluation results between different laboratories. Therefore, future research needs to focus on establishing standardized testing methods to accurately assess the long-term performance of self-cleaning surfaces and their feasibility in practical applications.

Overall, self-cleaning surface technology has shown tremendous potential for application, but it still faces many challenges in practical use. Future research should continue to strive to improve the durability of materials, reduce production costs, and expand their functional applications to promote the widespread application of this technology across various industrial fields.

## Data availability

Data generated or analyzed during this study are provided in full within the published article.

## Author contributions

Data curation: Suqi Xue, funding acquisition: Shanglei Yang, methodology: Suqi Xue, Xiner Li, supervision: Qiubo Li, Bangguo Hu, Xiner Li, validation: Shanglei Yang, writing – original draft: Suqi Xue, writing – review & editing: Suqi Xue.

## Conflicts of interest

There are no conflicts to declare.
